# Health-related Quality of Life and Related Factors among Rural Residents in Cambodia

**Published:** 2017-03

**Authors:** Youngran YANG, Betty BEKEMEIER, Jongsan CHOI

**Affiliations:** 1. School of Nursing, Research Institute of Nursing Sciences, Chonbuk National University, Jeonju-si, Jeollabuk-do, Republic of Korea; 2. School of Nursing, University of Washington, Seattle, Washington, USA; 3. College of Agricultural Life Sciences, Chonbuk National University, Jeonju-si, Jeollabuk-do, Republic of Korea

## Dear Editor-in-Chief

Quality of life (QOL) is a universal, complex, and multidimensional concept used globally that encompasses the physical, psychological, social and spiritual dimensions of an individual’s current life circumstances (1). QOL should be evaluated as an individual’s culture and value systems in relation to his/her goals, expectations, standards and concerns (2). In order to support health improvement and develop health promotion activities in a community or culture, an understanding the factors that determine good quality of life is necessary and integral to addressing health equality. Understanding these factors in rural communities and regions is particularly important, as HRQL tends to be poorer among residents of rural areas than among those living in urban environments (3); farmers, for example, have demonstrated a poorer quality of life when compared to other working groups (4).

Cambodia is primarily a rural, agrarian country, with 80% of its 14.68 million inhabitants living in rural regions, in addition, almost half (45.9%) of the country’s population lives in poverty (5). The study of rural residents’ HQRL should be conducted across cultural groups since HRQL can vary due to the diversity of residential environments, as well as the economic, social and cultural infrastructures that determine individual lifestyles (6). Until now, however, no apparent studies have been conducted to investigate HRQL and its related factors among the rural population in Cambodia.

This community-based cross-sectional survey was conducted to provide baseline data on HRQL and to understand its related factors among rural Cambodian residents, using a random sample of 223 people in the Krouch Chmar district in Kampong Cham, a province of Cambodia in 2015. The surveyed participants were adults, 18 yr of age and older, lived in the district for more than 5 yr. The study was structured using Andersen’s behavioral model, which depicts contextual characteristics, individual characteristics, and health behaviors affecting perceived health and evaluated health (7). HRQL was measured by the WHO Quality of Life-BREF questionnaire (WHOQOL-BREF) translated into Khmer (Cambodian language) by the International Society for Prosthetics and Orthotics (ISPO) for use in a multi-country study using WHO protocols; this questionnaire is composed of physical, psychological, social relationships, and environmental domains. Each domain in the WHOQOL-BREF survey has a score range of 4–20, with a higher score indicating better quality of life. Descriptive and multivariate regression analyses were employed.

The Chonbuk National University Institutional Review Board (IRB file No. 2015-06-031-002) approved the study protocol.

Among the four HRQL domains, the mean score for the social relationships domain was the highest (14.7). This was followed in descending order by the psychological (13.3), physical (12.8), and environmental (12.1) domains. Being 40 yr of age or over (*P*=0.036) or having over five members in the household (*P*=0.034) positively affected the physical domain (Table 1).

**Table 1:** Results of the multivariate regression analysis of the quality of life domains

**WHOQOL BREF domain**	**Variable**	**β**	***P***	***R^2^***
Physical domain	Age (≥40 yr)	−0.195	0.036	0.231
	Household size (≥5)	0.182	0.034	
	Self-perceived health (good)	0.227	0.025	
	Self-perceived happiness (happy)	0.243	0.011	
Psychological domain	Sex (female)	−0.181	0.038	0.390
	Age (≥40 yr)	−0.195	0.020	
	Never smoked	0.269	0.002	
	Self-perceived health (nor poor neither good)	0.222	0.020	
	Self-perceived health (good)	0.348	<0.001	
	Self-perceived happiness (happy)	0.243	0.005	
Social relationships	Spouse (Yes)	0.221	0.010	0.154
Environmental domain	Sex (female)	−0.294	0.002	0.286
	Never drank	0.188	0.047	
	Self-perceived health (good)	0.205	0.035	
	Self-perceived happiness (happy)	0.310	0.001	
Global score of quality of life	Disease (no)	0.166	0.041	0.394
	Self-perceived health (nor poor neither good)	0.302	0.001	
	Self-perceived health (good)	0.286	0.002	
	Self-perceived happiness (happy)	0.279	0.001	

Individuals never smoked had significantly higher mean scores than occasional smokers in the psychological domain (*P*=0.002) and being female (*P*=0.038) or 40 yr old or over (*P*=0.020) were negatively associated with the psychological domain. Residents currently living with a spouse had better HRQL in the social relationships domain (*P*=0.010). Being female (*P*=0.002) was associated with a lower mean score in the environmental domain. Participants who had no diseases ranked higher in the global QOL score (*P*=0.041). Individuals who perceived their health as good or themselves as happy had a positive association with all domains except social relationships. The majority of the rural residents in Cambodia experience a moderate level of HRQL.

The establishment of community health services that focus first on the effective access to health services should help the rural residents meet their most urgent needs regarding primary care access. Health care providers, social workers, and policymakers need to pay serious attention towards increasing the HRQL of marginalized people (females, that age 40 or over, or those who self-perceive as unhealthy and unhappy) as well as targeting the enhancement of the individual’s subjective happiness in an agricultural community. This study informs health priorities and strategies for HRQL improvement in rural areas of Cambodia and other similar cultures.
